# Peripheral Vestibular Dysfunction and Postural Control Impairments in Adolescents with Idiopathic Scoliosis: A Multimodal Clinical and Instrumental Analysis

**DOI:** 10.3390/medicina62061067

**Published:** 2026-05-31

**Authors:** Liliana Vlădăreanu, Elena Amaricai, Mihaela Minea, Elena Danteș, Iulia Tania Andronache, Mădălina Gabriela Iliescu

**Affiliations:** 1 Department of Rehabilitation, Faculty of Medicine, Campus-Corp B, Ovidius University of Constanța, 900470 Constanța, Romania; liliana.vladareanu@365.univ-ovidius.ro (L.V.); madalina.iliescu@365.univ-ovidius.ro (M.G.I.); 2 Department of Rehabilitation, Physical Medicine and Rheumatology, Research Center for Assessment of Human Motion, Functionality and Disability, “Victor Babeș” University of Medicine and Pharmacy, 300041 Timișoara, Romania; 3 Medical Doctoral School, Faculty of Medicine, Campus-Corp B, Ovidius University of Constanța, 900470 Constanța, Romania; mihaeala.minea@365.univ-ovidius.ro (M.M.); elena.dantes@365.univ-ovidius.ro (E.D.); 4 Department of Pneumology, Faculty of Medicine, Campus-Corp B, Ovidius University of Constanța, 900470 Constanța, Romania; 5 Internal Medicine Department, “Alexandru Gafencu” Emergency Military Hospital of Constanța, 900527 Constanța, Romania; andronacheiulia@gmail.com

**Keywords:** idiopathic scoliosis, vestibular dysfunction, major curve Cobb angle, balance, Fukuda Stepping Test, video-nystagmography, Romberg test, postural control

## Abstract

*Background and Objectives*: Idiopathic scoliosis (IS) has been conceptualized as a structural spinal deformity; emerging evidence suggests that postural control and vestibular mechanisms may contribute to curve development and functional severity. This study investigated the relationship between radiological parameters, postural stability, and vestibular dysfunction in adolescents with IS. *Materials and Methods*: A retrospective cohort of 177 patients aged 8–22 years was analyzed between 2022 and 2024. Standard radiography was performed on 135 participants to evaluate the major curve as established by the Cobb method, Nash–Moe classification, and Risser stage. Peripheral vestibular syndrome (PVS) was investigated using the Fukuda (FST), video-nystagmography (VNG), and instrumental Romberg tests on a stable and unstable platform. Associations between vestibular variables and radiographic parameters were explored using Mann–Whitney U and Kruskal–Wallis tests, supported by non-parametric correlations. *Results*: Female participants (63%) exhibited significantly higher initial major curve angle value compared with males (median 14° vs. 10.5°, *p* = 0.004). Positive FTS findings and the presence of peripheral vestibular syndrome were strongly associated with higher baseline and final major curve angles (both *p* < 0.001). Romberg performance showed significant correlations with major curve angle across stable and unstable conditions (r = 0.298–0.396, all *p* < 0.001). VNG identified multi-canal vestibular involvement, particularly anterior–horizontal combinations on the right ear, as being associated with substantially greater curve magnitude; left-ear impairment demonstrated similar non-significant trends. Curve localization did not differ by vestibular involvement. *Conclusions*: Patients with idiopathic scoliosis (IS) display consistent associations between vestibular dysfunction, impaired postural control, and greater curve severity. These findings support the clinical relevance of vestibular assessment in scoliosis evaluation and suggest a potential role for sensorimotor rehabilitation strategies. Integrating vestibular screening into standard care may enhance risk stratification and inform the clinician on individualized conservative management.

## 1. Introduction

Idiopathic scoliosis (IS) is defined as a non-physiological curvature in the frontal plane of the spine, by convention with a major curve Cobb angle value over 10°, with a non-identifiable cause [1,2,3].

Recent inquiries into the neurological origins of IS have suggested a potential connection between deficits in the vestibular system and the curvature of scoliosis, although this link has not been definitively proven [4,5,6,7,8,9,10,11,12,13]. The theory proposes that individuals with IS may have a higher likelihood of experiencing issues with their peripheral vestibular function compared to those in control groups. The vestibular system plays a crucial role in spatial orientation, encompassing both still and moving positioning, as well as the perception of verticality, sideways movement, and rotation. If there are inaccuracies in vertical perception early on in motor development due to problems with the peripheral vestibular system, this raises uncertainty about whether normal spinal alignment can be maintained during periods of rapid skeletal growth [4,5,6,7,8,9,10,11,12,13]. These theories compete with and/or complete another theory related to a neuroanatomy defect in patients with IS [14,15,16,17].

Motion sickness, known as kinetosis, is often connected to a heightened sensitivity in either one or both sides of the vestibular system and can manifest in children as young as one year old. Dizziness tends to be the primary symptom in children under six, with nausea becoming more pronounced later in life [18,19,20,21,22,23,24]. It is yet to be determined whether motion sickness, serving as an indication of peripheral vestibular issues, could potentially predict an increased likelihood of developing scoliosis or a more severe major curve angle over time.

The Fukuda Stepping Test (FST), which stems from Untenberger’s test from 1938 and was further developed by Fukuda in 1959, presents a straightforward clinical approach to assessing vestibular problems. By having the patient stand with eyes closed and taking 50 to 100 steps in place, a rotation exceeding 45° to either side suggests a positive outcome or indicates the affected side. The extent of rotation or forward movement aligns with the severity of symptoms [25,26,27,28].

As there are no confirmed connections between scoliosis screenings and a background of motion sickness or vestibular issues, our aim was to explore if a notable portion of children being assessed for scoliotic curves displayed a history of motion sickness and a positive FST result. Additionally, we delved into whether radiographic findings hinted at potential correlations between the major curve angle given by Cobb method measurements, motion sickness backgrounds, instrumental modified values for the Romberg test or for video-nystagmography (VNG), and/or positive FST outcomes.

The aims of the study included the following:

- Evaluating the potential relationship between the magnitude of major curve angles and peripheral vestibular issues;
- Exploring possible links between a history of motion sickness and vestibular dysfunction;
- Investigating associations between instrumental vestibular evaluations (such as Romberg tests and video-nystagmography) and the clinical and imaging characteristics of IS.

Moreover, the demographic and physical traits of the cohort were subject to statistical analysis as well.

## 2. Materials and Methods

### 2.1. Ethical Approval

All protocols implemented in the study were in accordance with ethical guidelines established by the institution and international standards. Each participant granted written consent for medical procedures and the use of clinical data for research. Participants who were 16 years old and above provided consent autonomously, whereas those under 16 needed approvals from both the individual and a parent or guardian. The research study was granted ethical approval by the Ethics Committee at the institution (Ethics Form No. 3739/11 March 2022).

### 2.2. Participants

During a 31-month duration from 15 March 2022 to 1 December 2024, a comprehensive assessment was conducted on 723 individuals. Among them, 177 minors aged 18-years of age and younger, including 106 females and 61 males, were selected based on specific criteria. These participants sought evaluation and treatment for developmental spinal conditions at the clinic for a period of 18 months. The ability to walk independently was a prerequisite for their involvement in the study to accommodate the FST within the clinical framework.

### 2.3. Study Design and Procedures

This study used a retrospective observational design. Out of the 723 patients examined across two clinical units, 177 met the inclusion criteria, and 135 gave their consent for standard radiographic evaluation.

#### 2.3.1. Inclusion Criteria

- Aged under 18 at enrolment;
- Idiopathic scoliosis (IS) as primary diagnosis;
- Independent ambulation—negative neurological history and no other medical condition with gait impairment;
- Written consent for scientific use of clinical data.

#### 2.3.2. Exclusion Criteria

- Age above 18 at enrolment;
- Prior diagnosis of neurological, congenital genetic, or post-traumatic orthopedic pathologies;
- Assisted gait that requires devices or caregiver support;
- Lack of written consent for the scientific use of clinical and gait/vestibular-related data.

#### 2.3.3. Standard Clinical and Instrumental Evaluation

- Fukuda Stepping Test (FST) [25,28];
- Adam’s forward bend test, performed both standing and seated [29,30];
- Finger-to-Toe distance (FTD), measured standing and supine (cm) [30];
- Video-nystagmography (VNG) taken on the rotatory chair at 2 rotations per second, towards the left and the right, head straight, tilted forward, to the left and to the right at full range of motion (ROM)—VNG was recorded using the Framinal platform goggles that continuously capture and quantify eye movements using minimal fixation; the recorded movements were evaluated with the platforms’ specialized software with respect to duration, direction, velocity and frequency, cut-off at <0.7%—as per Barany Society criteria;
- Instrumental Romberg test on the Framinal Multitest Equilibre platform, stable and unstable platform, eyes closed—two 30 s measurements were done while the patient was standing up on the Framinal platform with closed eyes, one with the platform locked in place, the second with the platform unlocked, both relying the patients’ sway—which was measured in mm^2^.

The FST and the instrumental evaluation were administered in a controlled, quiet environment. The patients were instructed to march in place for 50 steps with their eyes closed, beginning from a fixed-floor reference point. The procedure was demonstrated beforehand to ensure comprehension. Any opening of the eyes resulted in termination and repetition of the test. A positive result was defined as rotational deviation > 45° or forward displacement over 50 cm. Patients with positive FTS findings were referred for instrumental vestibular assessment using the Framinal Multitest Equilibre platform, which provided objective confirmation of the peripheral vestibular involvement and identified the affected side and semicircular canal(s).

#### 2.3.4. Standard Medical History Using a Structured Anamnesis Including

- History of motion sickness, classified as positive or negative according to Barany Society criteria [31];
- Dioptric correction status (emmetropia, hyperopia, myopia, astigmatism);
- Personal medical history relevant to the exclusion criteria.

#### 2.3.5. Radiological Assessment Using International Society on Scoliosis Orthopedic and Rehabilitation Treatment (SOSORT) Guidelines Standard [1]

- Full-spine cervical-thoraco-lumbar standing stitched radiographs in postero-anterior and lateral–lateral views;
- Major curve angle using the Cobb method (°): over 10° indicating scoliosis;
- Nash–Moe vertebral rotation grading 0–4;
- Risser sign: 0–5, indicating iliac crest ossification;
- Presence of congenital spinal malformations—only clinically insignificant spina bifida occulta was permitted [1].

All patients evaluated for this study only underwent conservative management for their scoliosis, using one of the physiotherapeutic scoliosis-specific exercise (PSSE) schools mentioned in the SOSORT guidelines None underwent surgery for their scoliotic curvatures [32,33,34,35,36,37,38,39].

#### 2.3.6. Statistical Analysis

Statistical analyses were performed using IBM SPSS Statistics v25, supplemented by Microsoft Excel and Word 2024. Quantitative variable distribution was assessed using the Shapiro–Wilk test. Depending on distribution, quantitative variables were expressed as mean ± standard deviation or as median with interpercentile range. Categorical variables were summarized as absolute and relative frequencies. Group comparisons for categorical data employed Fisher’s exact test due to its suitability for small sample sizes. Comparisons of independent non-parametric quantitative variables were conducted using the Mann–Whitney U test or Kruskal–Wallis H test. Significant Kruskal–Wallis results were followed by Dunn–Bonferroni post hoc analyses. Correlations between non-parametric quantitative variables were evaluated using Spearman’s rho. Paired non-parametric data were analyzed using the Wilcoxon signed-rank test. A receiver operating characteristic (ROC) curve based on the Cobb angle was generated to evaluate its predictive accuracy for peripheral vestibular syndrome. Diagnostic performance was reported as area under the curve (AUC) with 95% confidence intervals. Cutoff values were derived using the Youden Index. The statistical significance threshold was set at α = 0.05.

## 3. Results

### 3.1. Demographic and Age-Related Data

The analysis of the study cohort revealed that most participants were female, 85 (63%), with a mean age of 13.4 ± 2.8 years and a median age of 13 years (Table 1). Of the 177 patients included in this retrospective investigation, 135 consented to standard radiography in accordance with SOSORT recommendations for evaluating the Cobb angle, Nash–Moe classification, and Risser score. The mean value of the Cobb angle across the group evaluated radiographically was 16.1 ± 11.3° (median = 12°, IQR = 10–17). Based on the major curve–Cobb angle-derived scoliosis classification for severity, 76,27% of patients met the criteria for scoliosis, the majority presenting with grade I deformity.

The predominance of female patients (63%) aligns with existing epidemiological evidence reporting higher idiopathic scoliosis prevalence in girls.

**Table 1:** Demographic data.

Parameter	n	Value
Total sample size	177	—
Sex—Female	85	63%
Sex—Male	50	37%
Age (years)	135	Mean 13.4 ± 2.8 Median 13 (11–15) Range 8–22
Environment—Urban	122	90.4%
Environment—Rural	13	9.6%

Note: values represent demographic characteristics of the study group.

The initial major curve angle exhibited a non-parametric distribution in both sexes (Shapiro–Wilk *p* < 0.05), but, despite this, the final major curve angle did not differ significantly between males and females (Mann–Whitney U, *p* = 0.094). A statistically significant difference was identified (Mann–Whitney U, *p* = 0.004): female patients presented with a higher median initial major curve angle (14°, IQR 10–19.5) compared with males (10.5°, IQR 9–14) (Table 2).

The initial Nash–Moe index differed significantly between sexes (Mann–Whitney U, *p* = 0.037), with female patients showing higher values (median = 1, IQR 1–2) compared with male patients (median = 1, IQR 1–1.25), but no statistically significant differences were identified between sexes in the final Nash–Moe index (*p* = 0.527) (Table 2).

**Table 2:** Evolution of radiological and clinical parameters according to gender.

Parameter	Sex	Mean ± SD	Median (IQR)	Mean Rank	*p*-Value
Major curve Angle—Initial (°)	Female	17.2 ± 11.3	14 (10–19.5)	75.3	0.004
Major curve Angle—Initial (°)	Male	14.2 ± 11.1	10.5 (9–14)	55.5	
Major curve Angle—Final (°)	Female	16.0 ± 10.4	12 (10–19.5)	72.2	0.094
Major curve Angle—Final (°)	Male	14.0 ± 8.6	10.5(10–14.2)	60.7	
Nash–Moe—Initial	Female	1.4 ± 0.9	1 (1–2)	73.0	0.037
Nash–Moe—Initial	Male	1.1 ± 0.8	1 (1–1.2)	59.4	
Nash–Moe—Final	Female	1.2 ± 0.9	1 (0–2)	69.5	0.527
Nash–Moe—Final	Male	1.1 ± 0.8	1 (1–1)	65.4	
FTD—Initial	Female	20.3 ± 9.6	20 (15–25)	64.2	0.141
FTD—Initial	Male	22.6 ± 10.3	25 (15–30)	74.3	
FTD—Final	Female	1.9 ± 3.7	0 (0–5)	65.3	0.213 —
FTD—Final	Male	2.2 ± 2.7	0 (0–5)	72.5	

**Figure 1:**
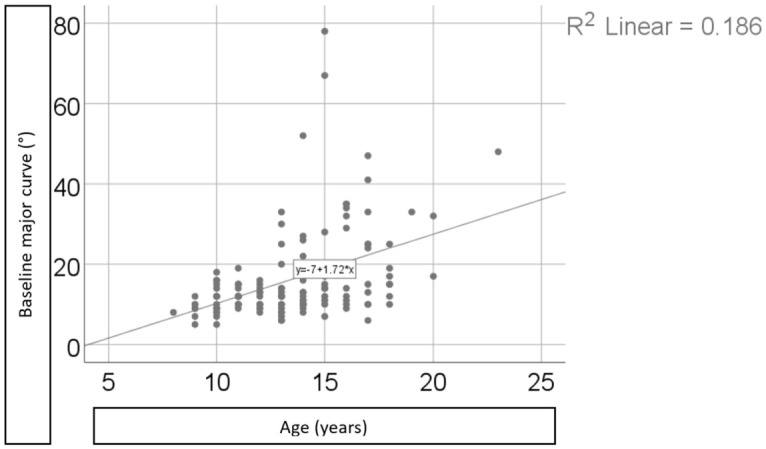
Correlation between baseline major curve value and age.

**Figure 2:**
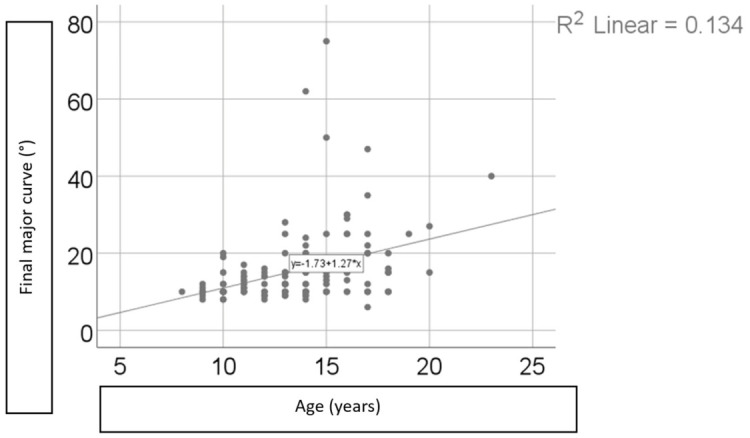
Correlation between final major curve value and age.

Regarding the association between age and major curve angle value at baseline and at final evaluation, as seen in Figure 1 and Figure 2, our batch data points show substantial variability, with a slight positive linear trend evident in each analysis, as older participants exhibiting marginally higher Cobb angles, although the wide dispersion of points underscores considerable inter-individual heterogeneity.

### 3.2. Test Results Related to Kinetosis, Peripheral Vestibular Dysfunction and Video-Nystagmography

The data collected between March 2022 and December 2024 were analyzed with respect to three primary hypotheses:

(a) The association between major curve angle value and FST results.
(b) Potential correlations between instrumental assessments of peripheral vestibular dysfunction (Romberg tests and video-nystagmography) and clinical and imaging characteristics of idiopathic scoliosis.

#### 3.2.1. Association Between Major Curve Angle Value and FST Result

**Figure 3:**
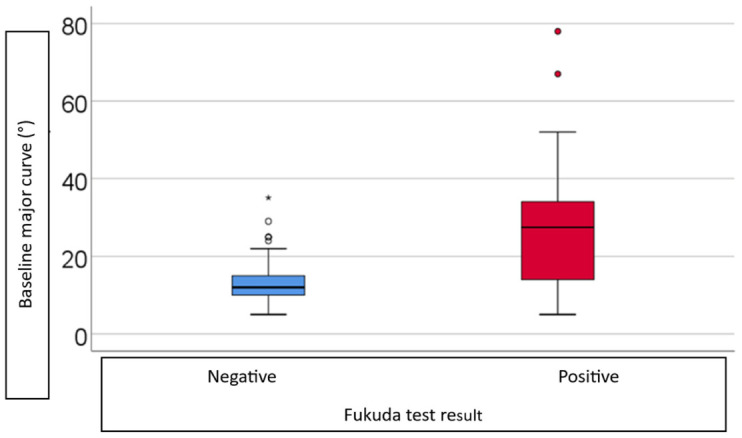
Baseline major curve value stratified by Fukuda test outcome. Open circles indicate outliers (>1.5 × IQR from the quartiles), and asterisks indicate extreme outliers (>3 × IQR).

In our lot the combined results from both datasets, as seen in Table 3 and Figure 3, consistently demonstrate clear differences between participants with negative and positive Fukuda test outcomes. In each dataset, individuals with negative results showed substantially lower mean values (12.7 ± 5.0 and 12.5 ± 3.9) and tightly clustered medians around 12, indicating relatively small deviations during the test. In contrast, participants with positive findings exhibited markedly higher mean values (28.2 ± 17.4 and 25 ± 16.2) and broader interquartile ranges, reflecting greater variability and larger deviations. The corresponding mean ranks follow the same pattern, with positive-test groups ranking considerably higher than negative-test groups across both datasets. All comparisons reached statistical significance, with *p*-values below 0.001 (and 0.016 in one case), reinforcing the robustness of the observed differences. Overall, the data consistently indicates that positive Fukuda test results are associated with substantially greater deviation than negative results, a pattern replicated across both measurement sets.

#### 3.2.2. Potential Correlations Between Instrumental Assessment of Peripheral Vestibular Dysfunction and Imaging Characteristics of Idiopathic Scoliosis

Across both datasets from Table 4, individuals exhibiting peripheral vestibular involvement demonstrated consistently higher symptom scores compared with those without vestibular pathology, with the most pronounced elevations observed in participants with unilateral right- or left-ear impairment. In both samples, the absence of peripheral vestibular syndrome was associated with markedly lower mean values and median distributions, findings supported by highly significant *p*-values (<0.001) that underscore the robustness of this contrast. By comparison, right-ear and left-ear groups showed substantially elevated mean ranks and broader variability, reflecting greater heterogeneity in symptom burden within these subgroups. Although the two datasets differed slightly in absolute values, most notably in the dispersion of scores within the right-ear category, the overall pattern remained stable, suggesting reproducibility across samples. Collectively, these results reinforce the association between peripheral vestibular dysfunction and increased symptom severity—meaning higher major curve angle value, while the consistency between datasets strengthens the reliability of these observations.

**Figure 4:**
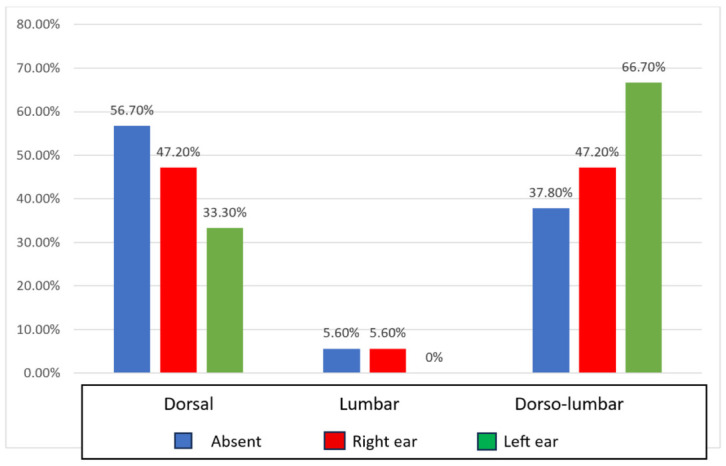
Patient distribution compared to scoliotic curve location and existence of PVS.

**Figure 5:**
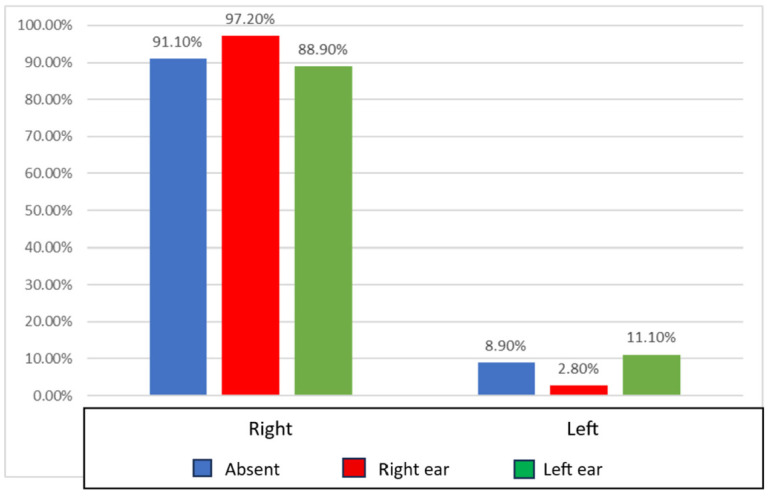
Patient distribution compared to scoliotic curve position and existence of PVS.

The data presented in Figure 4 and Figure 5 illustrates the patients’ distribution according to the location of the scoliotic curvature (dorsal, lumbar, dorsal–lumbar—determined by the existence of the vertebral rotation) and the lateralization of the curvature (right/left) and the presence of a PVS. Statistical analysis using Fisher’s test did not reveal significant differences between groups (*p* = 0.508 and *p* = 0.374), indicating that the frequency of the PVS does not vary significantly with respect to the localization of the scoliotic curvature.

**Figure 6:**
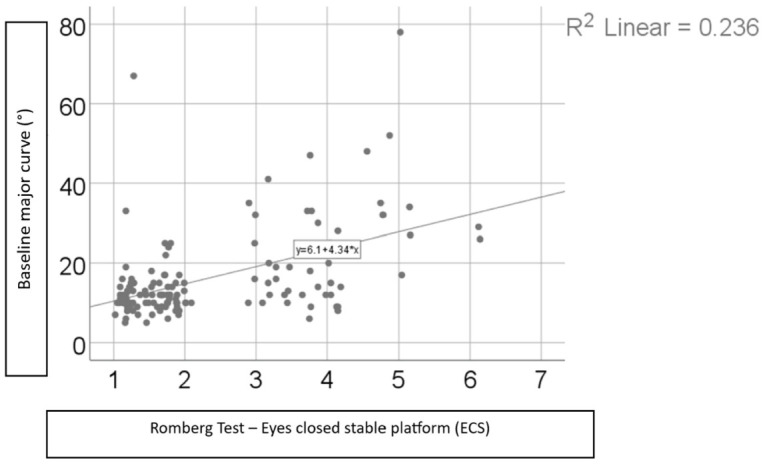
Correlation between baseline major curve value and Romberg test result for eyes closed stable (ECS) platform.

**Figure 7:**
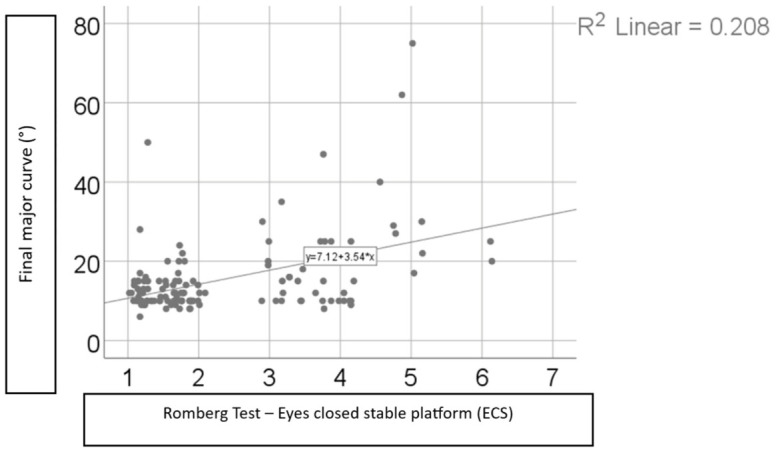
Correlation between final major curve angle value and Romberg test result for eyes closed stable (ECS) platform.

**Figure 8:**
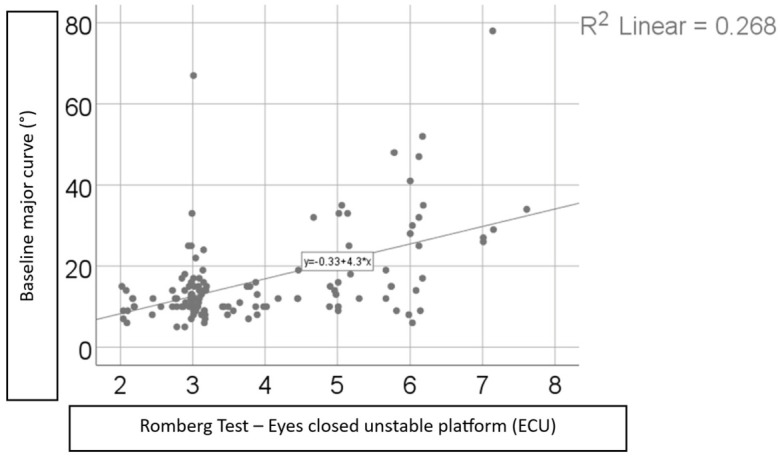
Correlation between baseline major curve angle value and Romberg test result for eyes closed unstable (ECU) platform.

**Figure 9:**
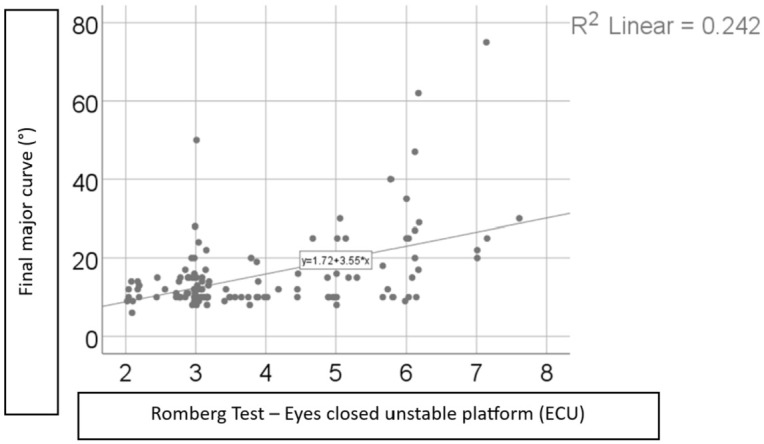
Correlation between final major curve angle value and Romberg test result for eyes closed unstable (ECU) platform.

The Framinal Multi-Equilibre platform used in our study determines two types of instrumental Romberg tests: eyes closed—stable platform (ECS), eyes closed—unstable platform (ECU). Both types of instrumental Romberg tests were correlated with the baseline and final values of the Cobb angle, as shown in Table 5 and Table 6 and Figure 6, Figure 7, Figure 8 and Figure 9.

Scatterplots from Figure 6, Figure 7, Figure 8 and Figure 9 reveal a consistent pattern: higher major curve angles are associated with progressively poorer balance control, with the relationship strengthening as postural demands increase. Compared with the stable platform condition, the unstable platform amplifies deficits, suggesting that individuals with more pronounced scoliosis may have reduced proprioceptive and vestibular efficiency, which becomes more evident under challenging sensory conditions.

These findings indicate that Romberg test performance—particularly on an unstable platform without visual feedback—may serve as a functional indicator of postural control impairment linked to scoliosis severity.

**Figure 10:**
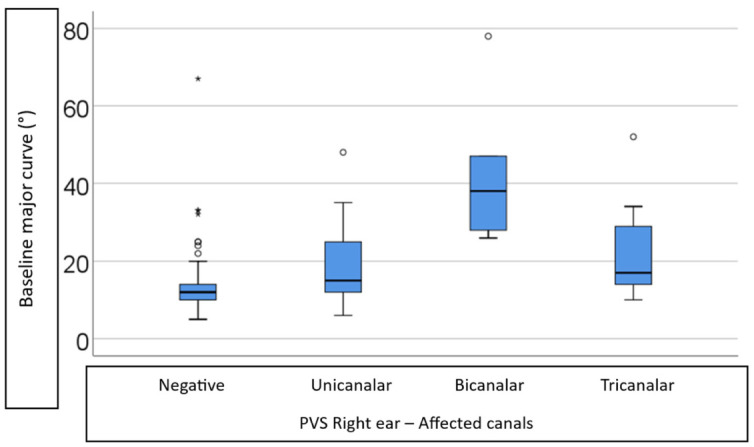
Baseline major curve angle value reported to the presence of right ear VPS and number of affected semicircular canals. Open circles indicate outliers (>1.5 × IQR from the quartiles), and asterisks indicate extreme outliers (>3 × IQR).

**Figure 11:**
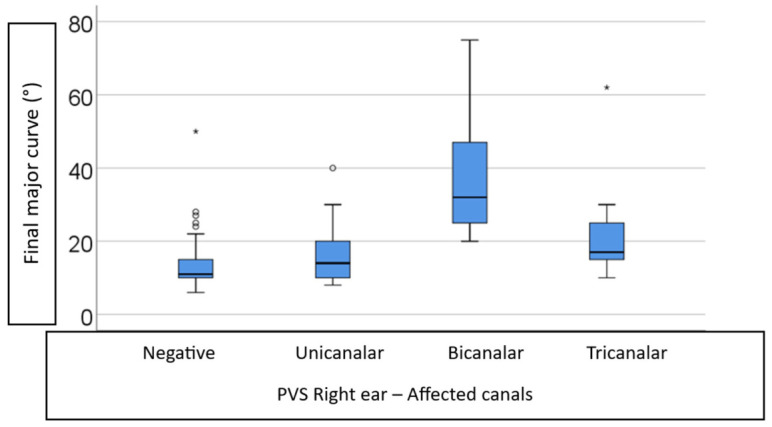
Final major curve angle Cobb value reported to the presence of right ear VPS and number of affected semicircular canals. Open circles indicate outliers (>1.5 × IQR from the quartiles), and asterisks indicate extreme outliers (>3 × IQR).

For the patients in the lot video-nystagmography was conducted for evaluation of right and left vestibular system, looking for a possible disorder for each semicircular canal. These findings were both compared with the value of baseline and final major curve angle value, as shown in Table 7 and Table 8, and in Figure 10 and Figure 11. There was no statistically significant association between baseline and final major curve angle values and left ear disturbance, as the number of patients affected on this side was too low.

Participants without vestibular involvement showed the lowest VNG values and consistently significant differences in both baseline and final assessments (*p* < 0.001). Multi-canal involvement, particularly anterior + horizontal, produced the highest deviations, whereas isolated anterior and horizontal canal involvement remained moderate and non-significant across time points. Posterior canal involvement showed significant differences in both assessments, indicating stable but meaningful deviation. Notably, three-canal involvement reached significance only at the final measurement (*p* = 0.008), suggesting a temporal change in vestibular response.

Video-nystagmography assessment of the left ear revealed variability in mean response amplitudes across impairment categories. Cases without impairment demonstrated relatively low mean values (15.8 ± 11.3 and 15.0 ± 9.9); whereas, combined canal involvement—particularly anterior + horizontal canal impairments—showed the highest mean and median responses (33 and 25, respectively), accompanied by markedly elevated mean ranks (125.0 and 121.5). Horizontal canal impairment also displayed increased median values (32 and 27) and higher mean ranks compared with most single-canal deficits. In contrast, posterior canal involvement consistently showed the lowest variability and smallest response magnitudes (12.5 ± 3.5 and 11.5 ± 0.7). The *p*-values reported for absent impairment and horizontal canal categories indicate that group differences do not consistently reach statistical significance, though the observed rank elevations in multi-canal and horizontal canal impairments suggest a trend toward greater functional disturbance in these subgroups.

When comparing the baseline major curve angle value with the presence of VPS on the right ear in connection with the number of affected semicircular canals—as seen in Figure 10—the distribution of the Cobb angle was non-parametric among unaffected patients, as indicated by the Shapiro–Wilk test (*p* < 0.05). Statistically significant differences were observed across groups according to the Kruskal–Wallis H test (*p* < 0.001). Post hoc Dunn–Bonferroni analyses showed that patients without right-ear involvement had a significantly lower initial Cobb angle (median = 12, IQR = 9.75–14.2) compared with those with single-canal (median = 15, IQR = 11.5–25; *p* = 0.039), two-canal (median = 38, IQR = 27.5–54.7; *p* < 0.001), or three-canal involvement (median = 17, IQR = 14–31.5; *p* = 0.011).

When evaluating the distribution of the final major curve Cobb angle value in relation to the existence of PVS value among patients without right-ear involvement was non-parametric, as indicated by the Shapiro–Wilk test (*p* < 0.05), see Figure 11. Group comparisons using the Kruskal–Wallis H test revealed statistically significant differences (*p* < 0.001). Post hoc Dunn–Bonferroni analyses showed that patients without right-ear involvement had a significantly lower initial Cobb angle (median = 11, IQR = 10–15) compared with those with two-canal involvement (median = 32, IQR = 23.75–54; *p* < 0.001) and three-canal involvement (median = 17, IQR = 12.5–27.5; *p* = 0.032). Additionally, the Cobb angle among patients with single-canal involvement (median = 14, IQR = 10–20) was significantly lower than that observed in patients with two-canal involvement (*p* = 0.021).

**Figure 12:**
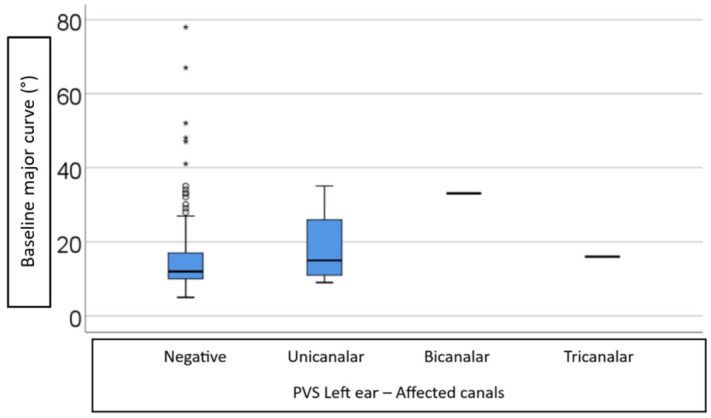
Baseline major curve angle value reported to the presence of left ear VPS and number of affected semicircular canals. Open circles indicate outliers (>1.5 × IQR from the quartiles), and asterisks indicate extreme outliers (>3 × IQR).

The data presented in Figure 12 shows the comparison between the initial major curve angle value and the number of affected canals identified through VNS in the left ear. The distribution of the major curve angle value was non-parametric among unaffected patients, as indicated by the Shapiro–Wilk test (*p* < 0.05). Group differences were not statistically significant according to the Kruskal–Wallis H test (*p* = 0.282), indicating that the initial major curve angle value did not differ significantly in relation to the number of affected canals in the left ear. Patients without vestibular involvement showed a median major curve angle value of 12 (IQR = 10–17) with a mean rank of 66.5. Those with single-canal involvement demonstrated a median value of 15 (IQR = 10–32) and a higher mean rank of 82.1, while patients with two-canal involvement exhibited the highest mean rank (125.0), with both mean and median values of 33. Individuals with three-canal involvement had a median Cobb angle of 16 and a mean rank of 97.5. Despite the numerical trend toward higher major curve angles values with increased canal involvement, these differences did not reach statistical significance.

**Figure 13:**
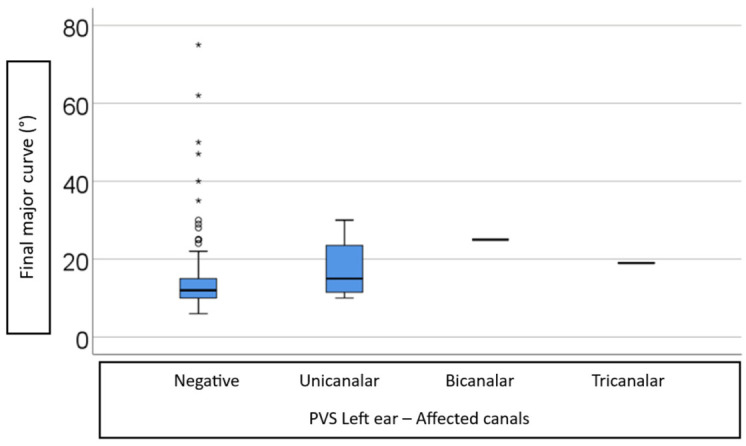
Final major curve angle value reported to the presence of left ear PVS and number of affected semicircular canals. Open circles indicate outliers (>1.5 × IQR from the quartiles), and asterisks indicate extreme outliers (>3 × IQR).

The data in Figure 13 summarizes the comparison of the final major curve angle value in relation to the number of affected canals identified through VNS in the left ear.

Among patients without vestibular involvement, the distribution of the major curve angle value was non-parametric according to the Shapiro–Wilk test (*p* < 0.05). Group comparisons using the Kruskal–Wallis H test revealed no statistically significant differences (*p* = 0.147), indicating that the final major curve angle value did not vary meaningfully with the degree of canal involvement. Patients without involvement showed a median major curve angle value of 12 (IQR = 10–15) and a mean rank of 66.1. Those with single-canal involvement demonstrated a median value of 15 (IQR = 11–27) and a higher mean rank of 88.7, while patients with two-canal involvement exhibited a median of 25 with a mean rank of 121.5. Individuals with three-canal involvement had a median value of 19 and a mean rank of 108.0. Although a numerical trend toward higher major curve angle values was observed with increasing canal involvement, these differences were not statistically significant.

## 4. Discussion

In this research, 177 adolescents were analyzed for IS to investigate demographic traits, radiological factors, and peripheral vestibular function. The study combined clinical, radiographic, and vestibular tests to explore a potential link between the severity of scoliosis and vestibular issues, adding to existing evidence on the subject. The results align with previous epidemiological studies and provide fresh perspectives on how peripheral vestibular problems and postural stability relate to IS.

### 4.1. Demographic Characteristics and Radiological Findings

The predominance of female participants 85 (63%) aligns with established epidemiological trends, which consistently report higher rates of IS in girls, particularly during early adolescence [40,41,42,43,44].

The mean age of 13.4 years is characteristic of the developmental window associated with scoliosis, its onset and progression risk. Although environmental distribution favored urban residence, environment was not a focal variable in this study.

Radiographic evaluation in 135 participants revealed a mean baseline major curve angle value of 16.1°, with most patients exhibiting mild (grade I) deformity. Despite non-normal distributions of baseline major curve angles in both sexes, female patients demonstrated significantly higher initial major curve angles and Nash–Moe indices than males. These results are consistent with prior findings that girls tend to present with greater curve severity. The absence of significant sex differences in final major curve angle and final Nash–Moe index may reflect comparable therapeutic responses or spontaneous stabilization of curves.

Age demonstrated only a weak positive association with major curve angle value at baseline and final evaluation. The wide dispersion of values suggests that age alone is insufficient for predicting curve magnitude and that other individual factors, including neurosensory function, may play a meaningful role.

### 4.2. Vestibular Function and Its Relationship to Scoliosis Severity

#### 4.2.1. Fukuda Stepping Test and Major Curve Cobb Angle

A consistent and robust association emerged between FST outcomes and major curve angle values. Participants with positive FST results—indicative of altered vestibulospinal integration—exhibited substantially higher baseline and final major curve angle values. Statistical significance was observed across all comparisons (*p* < 0.001 or *p* = 0.016), highlighting the reliability of this relationship. These findings suggest that vestibular asymmetry or dysfunction may be linked to the magnitude of spinal curvature.

#### 4.2.2. Peripheral Vestibular Disfunction and Major Curve Angle

Peripheral vestibular syndrome (PVS) demonstrated strong associations with increased major curve angle values. Participants with right or left-ear involvement consistently presented with greater scoliotic severity compared to those without vestibular deficits.

Right-ear involvement was particularly associated with statistically significant increases in baseline and final major curve angle values, whereas left-ear involvement showed similar numeric trends without reaching statistical significance—most likely due to fewer affected cases.

Curve localization (thoracic, lumbar, thoraco-lumbar) did not differ with PVS status, as indicated by nonsignificant Fisher’s exact test results. This finding suggests that vestibular dysfunction may influence overall curve magnitude rather than anatomical distribution of the deformity.

#### 4.2.3. Postural Stability and Instrumental Romberg Test Performance

Postural control, as assessed through instrumental Romberg tests (eyes closed on stable and unstable platform), correlated significantly with both initial and final major curve angle values. Stronger associations were observed under the unstable platform condition, indicating that increasing sensory and postural demands amplify deficits in patients with more severe scoliosis. These results support hypotheses in the published literature, that altered sensorimotor integration—particularly involving vestibular and proprioceptive inputs—may contribute to impaired postural regulation in IS and support the theory that postural instability—potentially arising from impaired multisensory integration—may contribute to the pathophysiology or progression of IS. At the same time, greater spinal deformity may compromise postural control through biomechanical mechanisms. Even if the directionality of this relationship is still uncertain, the present results underscore the potential utility of an instrumental Romberg assessment in identifying sensorimotor deficits relevant to scoliosis conservative management.

#### 4.2.4. Video-Nystagmography and Semicircular Canal Involvement

Video-nystagmography (VNG) revealed heterogenous canal involvement, with multi-canal dysfunction associated with the highest major curve angle values. For the right ear, combined anterior and horizontal canal impairment produced notably elevated major curve angles, significantly exceeding those observed in unaffected patients. Posterior canal involvement also showed significant associations. Left ear involvement followed similar trends but lacked statistical significance, as mentioned before, likely due to limited sample size.

These results align with emerging theories that vestibular dysfunction may influence axial alignment, tonic postural control, or asymmetric musculoskeletal loading, thereby contributing to scoliotic development and/or progression.

### 4.3. Clinical Implications

The results from this study have several implications for the clinical management of patients diagnosed with IS. The consistent associations identified between peripheral vestibular disfunction, balance impairment, and curve severity suggest that IS may encompass a boarder constellation of neurosensory abnormalities that extend beyond a structural spinal deformity alone. These findings reinforce the need to consider IS as a condition with multisystemic involvement, with potential consequences for long-term functional health.

The evidence of significant vestibular involvement—observed across dynamic vestibulospinal measures, postural stability testing, and VNS assessment—highlights the value of incorporating vestibular evaluation into routine scoliosis assessment. Simple clinical tools such as the FST, alongside more detailed balance assessments, may allow clinicians to identify adolescents with additional functional vulnerabilities that are not detectable through radiographic evaluation alone. Early identification of vestibular impairment may help predict which patients are at greater risk of functional instability or curve progression and may support more personalized care plans. Also further prospective interventional studies could be devised in order to determine if compensatory curves also depend or not on vestibular impairment, or if they are one of the aspects that can interfere with the results of the vestibular testing, resulting in false positivation.

The observed relationship between peripheral vestibular dysfunction and greater major curve angle value raises the possibility that targeted sensorimotor interventions could complement existing conservative treatments such as bracing and physiotherapeutic scoliosis-specific exercise (PSSE) [1]. Vestibular rehabilitation therapy, which is well established in the management of chronic vestibular disorders, may hold potential to improve postural stability and sensory integration in adolescents with IS. Although interventional research is required to confirm clinical benefits, integrating balance training and vestibular-focused rehabilitation into conservative scoliosis management may enhance functional outcomes, particularly in patients who exhibit significant sensorimotor deficits.

These findings underscore the importance of multidisciplinary approaches to IS conservative management. Collaboration among orthopedic clinicians, physiotherapists, otolaryngologists, neurologists, and rehabilitation specialists may enable more comprehensive evaluation and treatment, ensuring that vestibular contributions to balance dysfunction and postural asymmetry are appropriately addressed. Such an approach aligns with contemporary clinical models that emphasize functional assessment, early detection, and multimodal intervention in managing musculoskeletal conditions.

The broader clinical relevance of these results lies in their potential to shift aspects of scoliosis care from a reactive model—focused primarily on monitoring curve progression—to a more proactive strategy that integrates neurosensory assessment and targeted rehabilitation. This shift may improve long-term functional outcomes and quality of life for adolescents with IS by addressing modifiable factors that may contribute to postural control deficits or biomechanical asymmetry.

The present study supports the growing recognition that vestibular and postural abnormalities are important components of IS and may influence both functional capacity and curve severity. Incorporating vestibular evaluation and individualized sensorimotor rehabilitation strategies into standard of care pathways may offer meaningful therapeutic benefit for this patient population. However further prospective and interventional studies, clinical as well as radiographic, also incorporating sagittal plane evaluation are needed.

### 4.4. Study Limitations

There are several limitations to this study that should be acknowledged.

First, this was an observational retrospective study conducted from the framework of conservative scoliosis management so the findings should not be considered or interpreted as demonstrating causality or definitive treatment efficacy. The primary purpose of this study was to identify clinical associations and trends observed during routine conservative follow-up rather than to evaluate surgical indications or outcomes or major structural curve correction. In this study only age maturity was considered, as opposed to the Risser or Saunders osseous maturation classifications, as they were not available for the entirety of the studied batch. Also, another potential limitation for this study was that sagittal parameters were not specifically evaluated and incorporated in the clinical reasoning. In the current analysis, the main investigation was focused on the coronal plane major curve evaluation, as this constitutes the most known, evaluated and easily available parameter for all scoliotic patients. Nevertheless spinal imbalance may play an important role in postural adaptations and peripheral vestibular compensation mechanisms in scoliotic patients, eventually influencing both postural control and/or curve progression.

Second, the multifactorial nature of IS progression, including potential neurologic and hereditary influences, limits the ability to establish mechanistic conclusions from observational data alone.

Third, the study reflects the perspective and expertise of conservative treatment practice, which may differ from surgical or purely orthopedic interpretative frameworks.

## 5. Conclusions

The convergence of findings across multiple vestibular measures indicates for this batch of patients that peripheral vestibular dysfunction may be functionally relevant in adolescents with IS. Assessments such as the FTS, Romberg tests, and, when available, VNS could serve as adjunct diagnosis tools to identify patients with greater postural instability or potential risk of curvature progression.

Interventions targeting vestibular rehabilitation may represent a promising, though underexplored, component of conservative scoliosis management in physiotherapeutic scoliosis-specific exercise programs (PSSE).

More research, involving both prospective interventional and multicentered studies, should be conducted to determine the clinical benefits for such therapeutic exercises regarding AIS patient outcomes in terms of curvature progression. These studies should cover different scoliotic curvature classifications systems and/or patterns, such as Lenke.

## Figures and Tables

**Table 3:** Association between the result for the FST and initial and final Cobb values.

Fukuda Stepping Test Result	Mean ± SD	Median (IQR)	Mean Rank	*p* *
Negative—initial major curve (*p* < 0.001 **)	12.7 ± 5.0	12 (10–15)	58.4	<0.001
Positive—initial major curve (*p* = 0.016 **)	28.2 ± 17.4	27.5 (14–34.2)	101.3	
Negative—final major curve (*p* < 0.001 **)	12.5 ± 3.9	12 (10–15)	59.7	<0.001
Positive—final major curve (*p* < 0.001 **)	25 ± 16.2	23.5 (10.7–30)	96.9	

Note: Mann–Whitney U test (*) used for sex comparisons. Normality assessed using the Shapiro–Wilk test (**).

**Table 4:** Comparison between initial/final major curve angle value and the existence of peripheral vestibular syndrome (PVS).

Peripheral Vestibular Syndrome/Initial Major Curve Angle Value	Mean ± SD	Median (IQR)	Mean Rank	*p* *
Absent (*p* < 0.001 **)	12.6 ± 7.3	11.5 (9.7–14)	56.0	<0.001
Right ear (*p* < 0.001 **)	23.2 ± 15.4	18.5 (12–31.5)	90.4	
Left ear (*p* = 0.281 **)	22.2 ± 9.8	20 (13.5–32.5)	97.5	
Absent (*p* < 0.001 **)	12.5 ± 5.3	11 (10–14)	57.8	<0.001
Right ear (*p* < 0.001 **)	20.9 ± 15.0	16 (10–25)	84.9	
Left ear (*p* = 0.680 **)	20.3 ± 6.9	20 (13.5–26)	101.5	

* Mann–Whitney U Test, ** Shapiro–Wilk Test.

**Table 5:** Correlation between initial/final major curve angle value and Romberg test result for eyes closed stable (ECS) platform.

Correlation Pair	*p*-Value	Correlation Coefficient (R)
Initial major curve Angle × ECS	*p* < 0.001 ^∗∗^	R = 0.396
Final major curve Angle × ECS	*p* < 0.001 **	R = 0.298

Notes: Statistically significant correlations are marked with **. Values reflect non-parametric correlation analysis.

**Table 6:** Correlation between initial/final major curve angle value and Romberg test result for eyes closed unstable (ECU) platform.

Correlation Pair	*p*-Value	Correlation Coefficient (R)
Initial Cobb Angle × ECU	*p* < 0.001 **	R = 0.390
Final Cobb Angle × ECU	*p* < 0.001 **	R = 0.307

Notes: Statistically significant correlations are marked with **. Values reflect non-parametric correlation analysis.

**Table 7:** Baseline/final Cobb angle value reported to video-nystagmography for the right ear.

Right Ear	Canal Involvement	Mean ± SD	Median (IQR)	Mean Rank	*p* *
Baseline major curve angle	Absent (*p* < 0.001 **)	13.1 ± 7.8	12 (10–14)	57.5	<0.001
	Anterior canal (*p* = 0.304 **)	13.5 ± 4	12.5 (10–18)	68.4	
	Anterior + horizontal canal (*p* = **)	62.5 ± 21.9	62.5 (47–78)	133.0	
	Three-canal involvement (*p* = 0.124 **)	23.6 ± 13.3	17 (14–29)	100.0	
	Anterior + posterior canal (*p* = **)	28	28	119.0	
	Horizontal canal (*p* = 0.511 **)	23.3 ± 8.9	25 (15–32)	103.8	
	Horizontal + posterior canal (*p* = 0.780 **)	34 ± 7.5	35 (30.5–38)	125.1	
	Posterior canal (*p* = 0.037 **)	18.7 ± 15	12 (10.5–22)	69.6	
Final major curve angle	Absent (*p* < 0.001 **)	12.8 ± 5.6	11 (10–15)	59.4	<0.001
	Anterior canal (*p* = 0.062 **)	13 ± 3.9	11 (10–16)	63.5	
	Anterior + horizontal canal (*p* = **)	61 ± 19.8	61 (47–75)	133.5	
	Three-canal involvement (*p* = 0.008 **)	23 ± 16.0	17 (15–25)	96.8	
	Anterior + posterior canal (*p* = **)	25	25	121.5	
	Horizontal canal (*p* = 0.543 **)	18.7 ± 7.05	20 (14–25)	93.7	
	Horizontal + posterior canal (*p* = 0.780 **)	28 ± 7.5	29 (24.5–32)	123.0	
	Posterior canal (*p* = 0.025 **)	17 ± 11.5	12 (10–19.5)	68.5	

* Kruskal–Wallis H Test, ** Shapiro–Wilk Test.

**Table 8:** Baseline/final Cobb angle value reported to video-nystagmography for the left ear.

Left Ear	Impairment Type	Mean ± SD	Median (IQR)	Mean Rank	*p* *
Baseline major curve value	Absent (*p* < 0.001 **)	15.8 ± 11.3	12 (10–17)	66.5	0.350
	Anterior canal (*p* = **)	14.5 ± 7.7	14.5 (9–20)	65.7	—
	Anterior + horizontal canal (*p* = **)	33	33	125.0	—
	Tricanal (*p* = **)	16	16	97.5	—
	Horizontal canal (*p* = 0.230 **)	26.3 ± 12.5	32 (22–33.5)	104.6	—
	Posterior canal (*p* = **)	12.5 ± 3.5	12.5 (10–15)	64.7	—
Final major curve value	Absent (*p* < 0.001 **)	15.0 ± 9.9	12 (10–15)	66.1	0.156
	Anterior canal (*p* = **)	15 ± 7.0	15 (10–20)	73.7	—
	Anterior + horizontal canal (*p* = **)	25	25	121.5	—
	Tricanal (*p* = **)	19	19	108.0	—
	Horizontal canal (*p* = 0.363 **)	24 ± 7.9	27 (21–28.5)	115.6	—
	Posterior canal (*p* = **)	11.5 ± 0.7	11.5 (11–12)	63.5	—

* Kruskal–Wallis H Test, ** Shapiro–Wilk Test.

## Data Availability

Data and methodological support may be available upon reasonable and legally permissible request.

## Abbreviations

The following abbreviations are used in this manuscript:

AIS	Adolescent Idiopathic Scoliosis Multidisciplinary
AUC	Area Under the Curve
ECS	Eyes Closed Stable
ECU	Eyes Closed Unstable
EOS	Eyes Open Stable
FST	Fukuda Stepping Test
FTD	Finger-to-Toe Distance
IS	Idiopathic Scoliosis
PSSE	Physiotherapeutic Scoliosis-Specific Exercise
PVS	Peripheral Vestibular Syndrome
ROM	Range of Motion
SOSORT	International Society on Scoliosis Orthopedic and Rehabilitation Treatment
VNG	Video-nystagmography
